# Supplementary material for: Visualization of Type IV-A1 CRISPR-mediated repression of gene expression and plasmid replication
Source: Nucleic Acids Res. 2024 Oct 9;52(20):12592–603. doi: 10.1093/nar/gkae879 (PMC11551745; doi:10.1093/nar/gkae879)
Supplement: gkae879_Supplemental_File [file gkae879_supplemental_file.pdf]

Supplementary Data

Visualization of Type IV-A1 CRISPR-mediated repression of gene expression and plasmid replication

Mariana Sanchez-Londono[1†], Selina Rust[1†], Rogelio Hernández-Tamayo[2,3], José Vicente Gomes-Filho[1], Martin Thanbichler[1,2,3], and Lennart Randau*[1,3]

[1] Department of Biology, Philipps-Universität Marburg, Marburg, Germany
[2] Max Planck Institute for Terrestrial Microbiology, Marburg, Germany
[3] Center for Synthetic Microbiology (SYNMIKRO), Marburg, Germany

[†] These authors contributed equally

Supplementary data content

Supplementary Table S1. Diffusion constants of slow, intermediate, and mobile molecule populations of the native Type IV-A1-mNeonGreen crRNP complex in *P. oleovorans*.

Supplementary Table S2. Mean number of molecules of the recombinant Type IV-A1 crRNP complex present in the different conditions in *E. coli* BL21-AI:*dnaXmS*.

Supplementary Table S3. Diffusion constants of slow, intermediate, and mobile molecule populations of the recombinant Type IV-A1-mNeonGreen crRNP complex in *E. coli* BL21-AI: *dnaXmS*.

Supplementary Table S4. Diffusion constants of slow, intermediate, and mobile molecule populations of DnaX-mScarlet in *E. coli* BL21-AI:*dnaXmS* in the presence of recombinant Type IV-A1-mNeonGreen crRNP complex and target/non-target plasmids.

Supplementary Table S5. Plasmids used in this study.

Supplementary Table S6. Strains used in this study.

Supplementary Table S7. Spacers used for Type IV-A1 and dCas9 CRISPRi assays.

Supplementary Table S8. Primers used for RT-qPCR.

Supplementary Figure S1. RT-qPCR of different spacers tested for dCas9-mediated CRISPRi on *hisA* gene

Supplementary Figure S2. Analysis of CRISPRi transcriptome effects.

Supplementary Figure S3. Spatiotemporal dynamics of the mNeonGreen-tagged crRNPs with different targets.

Supplementary Figure S4. Spatiotemporal dynamics of the DnaX-mScarlet protein in cells expressing mNeonGreen-tagged crRNPs targeting or not targeting a plasmid.

Supplementary Table S1. Diffusion constants of slow, intermediate, and mobile molecule populations of the native Type IV-A1-mNeonGreen crRNP complex in *P. oleovorans*.

**Strain**	**cells***	**tracks**	**D** [a]	**D_1** [b]	**D_2** [c]	**D_3** [d]	**F_1** [a]	**F_2** [b]	**F_3** [c]
Wild type	98	3614	0.07 ± 0.011	0.023 ± 0.001	0.128 ± 0.002	0.487 ± 0.002	33 ± 1	36 ± 2	31 ± 1
Wild type + plasmid	107	2350	0.05 ± 0.018	0.023 ± 0.002	0.128 ± 0.002	0.487 ± 0.001	2 ± 1	63 ± 1	35 ± 2

*Cells analyzed here were obtained from a total of two independent biological replicates (colonies).

Da, MSD, average diffusion constant of all molecules ($\mu m^2 \cdot s^{-1}$).

D_1b, diffusion constant of slow population ($\mu m^2 \cdot s^{-1}$).

D_2c, diffusion constant of intermediate population ($\mu m^2 \cdot s^{-1}$).

D_3d, diffusion constant of mobile population ($\mu m^2 \cdot s^{-1}$).

F_1a, percentage of slow population (%)

F_2b, percentage of intermediate population (%)

F_3c, percentage of mobile population (%)

Supplementary Table S2. Mean number of molecules of the recombinant Type IV-A1 crRNP complex present in the different conditions in *E. coli* BL21-AI:*dnaXmS*.

**Strain**	**Mean molecules**
Genome target	58
Non-target plasmid	75
Target plasmid	83

Supplementary Table S3. Diffusion constants of slow, intermediate, and mobile molecule populations of the recombinant Type IV-A1-mNeonGreen crRNP complex in *E. coli* BL21-AI: *dnaXmS*.

Strain	cells*	tracks	D^{a}	D_1^{b}	D_2^{c}	D_3^{d}	F_1^{a}	F_2^{b}	F_3^{c}
Genome target	130	5723	0.11 ± 0.018	0.005 ± 0.002	0.06 ± 0.001	0.42± 0.002	23± 2	66± 2	11± 1
Non-target plasmid	89	2809	0.14 ± 0.015	0.005 ± 0.001	0.06 ± 0.002	0.42 ± 0.001	19± 1	68± 2	13± 2
Target plasmid	97	3039	0.13 ± 0.017	0.005 ± 0.001	0.06 ± 0.001	0.42 ± 0.002	31± 2	58± 2	11± 2

*Cells analyzed here were obtained from a total of three independent biological replicates (colonies).

D^{a}, MSD, average diffusion constant of all molecules ($\mu m^2 \cdot s^{-1}$).

D_1^{b}, diffusion constant of slow population ($\mu m^2 \cdot s^{-1}$).

D_2^{c}, diffusion constant of intermediate population ($\mu m^2 \cdot s^{-1}$).

D_3^{d}, diffusion constant of mobile population ($\mu m^2 \cdot s^{-1}$).

F_1^{a}, percentage of slow population (%)

F_2^{b}, percentage of intermediate population (%)

F_3^{c}, percentage of mobile population (%)

Supplementary Table S4. Diffusion constants of slow, intermediate, and mobile molecule populations of DnaX-mScarlet in *E. coli* BL21-AI:*dnaXmS* in the presence of recombinant Type IV-A1-mNeonGreen crRNP complex and target/non-target plasmids.

Strain	cells*	tracks	D^a	D_1^b	D_2^c	D_3^d	F_1^a	F_2^b	F_3^c
Non-target plasmid	89	1030	0.02± 0.01	0.024 ± 0.001	0.11± 0.01	0.88± 0.01	4± 1	50± 1	46± 2
Target plasmid	97	1100	0.01± 0.01	0.024 ± 0.001	0.11± 0.01	0.88± 0.02	10± 1	81± 2	9± 1

*Cells analyzed here were obtained from a total of three independent biological replicates (colonies).

D^a, MSD, average diffusion constant of all molecules ($\mu m^2 \cdot s^{-1}$).

D_1^b, diffusion constant of slow population ($\mu m^2 \cdot s^{-1}$).

D_2^c, diffusion constant of intermediate population ($\mu m^2 \cdot s^{-1}$).

D_3^d, diffusion constant of mobile population ($\mu m^2 \cdot s^{-1}$).

F_1^a, percentage of slow population (%)

F_2^b, percentage of intermediate population (%)

F_3^c, percentage of mobile population (%)

Supplementary Table S5. Plasmids used in this study.

Plasmid	Description	Features	Reference
pSIM5	Homologous recombination vector expressing λ-Red gam, exo, and bet	Cm^R; Temperature sensitive origin pSC101-ts	(Kovach et al., 1995)
pEMG	Suicide vector used for deletions and insertions in *P. oleovorans*	Km^R, oriT, traJ, lacZα, oriV(R6K);	(Martínez-García & Lorenzo, 2011)
pMSL15	pEMG derivative; homologious regions of *P. oleovorans* for generation of *P. oleovorans* ΔCRISPR		This study
pMSL35	pEMG derivative; homologious regions of P. oleovorans for generation of *P. oleovorans* Δ*dinG*		This study
pSR58	pEMG derivative; homologious regions and *sfgfp* with pBAD promoter for generation of *P. oleovorans* expressing sfGFP; *sfgfp* insertion site: between *tadA* and *mltF*		This study
pSEVA6213S	Helper plasmid for generation of insertion and deletion strains	Gm^R, oriV (RK2), Pem_7 Promoter, I-*SceI*	(Wirth et al., 2020)
pACYCDuet™-1	Generation of pSR14 and pSR15	Cm^R, p15A ori, 2x T7 promoter, *lacI*	Novagen
pCDFDuet™-1	Generation of pMSL26 and pSR56	Sm^R, 2x T7 promoter, CDF origin, *lacI*	Novagen
pETDuet™-1	Generation of pMSL13	Amp^R , 2x T7 promoter, pBR322 origin, *lacI*	Novagen
pRSFDuet™-1	Generation of pSR77	Km^R, 2x P_{T7}, T7 terminator, RSF origin, *lacI*	Novagen
pSR77	pRSFDuet™-1 derivative; MCS1: Type IV-A1 CRISPR-Cas from *P.oleovorans* (*csf5, csf1, csf2, csf3*); MCS2: *csf4;* repeat-spacer-repeat fragment with BseRI restriction site on the spacer. Use as Type IV-A1 negative control in the histidine auxotrophs CRISPRi assays		This study

Plasmid	Description	Features	Reference
pMSL26	pCDFDuet™-1 derivative; MCS1: dCas9 synthetic, T7 promoter, sgRNA containing spacer and tracrRNA; BsaI restriction enzyme on spacer. Use as dCas9 negative control in the histidine auxotrophs CRISPRi assays		This study
pMSL41	pSR77 derivative; spacer targets *hisA* in *E. coli* BL21-AI		This study
pMSL45	pSR77 derivative; spacer targets *hisA* coding strand in *E. coli* BL21-AI		This study
pMSL46	pSR77 derivative; spacer targets promoter of *his operon* in *E. coli* BL21-AI		This study
pMSL47	pSR77 derivative; spacer targets internal promoter in *hisC* in *E. coli* BL21-AI		This study
pMSL44	pMSL26 derivative; spacer targets *hisA* in *E. coli* BL21-AI		
pMSL55	pMSL26 derivative; spacer targets *hisA* in *E. coli* BL21-AI		This study
pMSL56	pMSL26 derivative; spacer targets *hisA* in *E. coli* BL21-AI		This study
pMSL57	pMSL26 derivative; spacer targets *hisA* in *E. coli* BL21-AI		This study
pMSL70	pMSL26 derivative; spacer targets *hisA* in *E. coli* BL21-AI		This study
pMSL13	pETDuet™-1 derivative; MCS1: Type IV-A1 CRISPR-Cas from *P.oleovorans* (*csf5, csf1, csf2, csf3*); MCS2: *csf4;*		This study
pSR56	pCDF-Duet™-1 derivative; MCS1: Type IV-A1 repeat-spacer-repeat fragment with BseRI restriction site on the spacer.		This study
pSR54	pSR56 derivative; spacer targets *lacZ* in *E. coli* BL21-AI; for genome targeting assays using the recombinant system		This study
pSR66	pSR13 derivative; MCS1: Type IV-A1 CRISPR-Cas from *P.oleovorans* (*csf5*, ***csf1:mNeonGreen***, *csf2*, *csf3*); MCS2: *csf4;*		This study
pSR24	pSR56 derivative; spacer targets sequence on pSR14		
pSR14	pACYCDuet™-1 derivative; MCS1: 5′ AAG 3′ PAM-protospacer type IV; Target plasmid in the SMM assays.		(Guo et al., 2022)
pSR15	pACYC-Duet™-1 derivative, MCS1: random 32 nt sequence; Non-target plasmid in the SMM assays.		(Guo et al., 2022)

**Plasmid**	**Description**	**Features**	**Reference**
pS448	Generation of pMSL17. Derivative of pSEVA448 used for CRISPR-Cas9 counterselection; cured of BsaI restriction sites.	Sm^R, oriV (pRO1600), Pm→Cas9, XylS/Pem7 promoter →sgRNA	(Wirth et al., 2020)
pMSL17	pS448 derivative; sgRNA targeting Kanamycin gene from pEMG vector		This study
pSEVA424	Generation of pSR106	Sm^R, oriV (pRO1600), *lacI*	(Silva-Rocha et al., 2013)
pSR106	pSEVA424 derivative; insertion of *araC* and Type IV-A1 repeat-spacer-repeat fragment with BseRI restriction sites in the spacer		This study
pSR112	pSR106 derivative; carrying spacer against *sfgfp* in *P. oleovorans*		This study
pSR121	pSR106 derivative; carrying spacer against promoter of *sfgfp* in *P. oleovorans*		This study
pSR122	pSR106 derivative; carrying spacer against *sfgfp* in *P. oleovorans*		This study
pSR124	pSR106 derivative; carrying spacer against protospacer ~ 500 bp upstream of mid of *sfgfp* in *P. oleovorans*		This study
pSR125	pSR106 derivative; carrying spacer against protospacer ~ 400 bp downstream of mid of *sfgfp* in *P. oleovorans*		This study
pSR129	pSR106 derivative; carrying spacer against protospacer ~ 2.8 kb upstream of mid of *sfgfp* in *P. oleovorans*		This study
pSR150	pSR106 derivative; carrying spacer against protospacer ~ 900 bp upstream of mid of *sfgfp* in *P. oleovorans*		This study
pSR151	pSR106 derivative; carrying spacer against protospacer ~ 900 bp upstream of mid of *sfgfp* in *P. oleovorans*		This study
pSR152	pSR106 derivative; carrying spacer against protospacer ~ 800 bp downstream of mid of *sfgfp* in *P. oleovorans*		This study
pSR153	pSR106 derivative; carrying spacer against protospacer ~ 800 bp downstream of mid of *sfgfp* in *P. oleovorans*		This study
pSR154	pSR106 derivative; carrying spacer against protospacer ~ 1.6 kb upstream of mid of *sfgfp* in *P. oleovorans*		This study

Plasmid	Description	Features	Reference
pSR155	pSR106 derivative; carrying spacer against sequence ~88 kb upstream of mid of *sfgfp* in *P. oleovorans*		This study
pSR160	pSR106 derivative; carrying spacer against protospacer ~ 2.8 kb upstream of mid of *sfgfp* in *P. oleovorans*		This study
pSR162	pSR106 derivative; carrying spacer against protospacer ~ 3.8 kb upstream of mid of *sfgfp* in *P. oleovorans*		This study

Supplementary Table S6. Strains used in this study.

Strain	Feature	Reference
Pseudomonas oleovorans DSM1045	Wild type	Leibniz Institute DSMZ
P. oleovorans DSM1045:*sfgfp*	Wild type expressing sfGFP	This study
P. oleovoransΔCRISPRarray:sfgfp	Δ*TypeIV-A1-CRISPRarray* expressing sfGFP	This study
P. oleovoransΔCasDinG:sfgfp	Δ*CasDinG* expressing sfGFP	This study
Pseudomonas oleovorans:csf5mNeongreen	*csf5-mNeongreen*	(Guo et al., 2022)
E.coli BL21-AI	F– ompT gal dcm lon hsdSB(rB–mB–) [malB+]K-12(λS) araB::T7RNAP-tetA	Thermo Fisher
BL21-AI:*dnaX-mS*	*dnaX-mScarlet*, Km^R	This study

Supplementary Table S7. Spacers used for Type IV-A1 and dCas9 CRISPRi assays.

Plasmid	Description	Spacer sequence (5′-3′)	Restriction recognition sites for insert
pMSL26	Negative control used in the histidine auxotrophic dCas9 CRISPRi assays	GAGACCCGAGACTGGTCTCA	BsaI
pMSL41	Type IV-A1 crRNP; Protospacer is located on the non-coding strand of *hisA;* PAM: 5′-AAG-3′	TGTTGCACCTGGTGGATCTGACCGGGGCAAAA	NA
pMSL44	dCas9; Protospacer is located on the non-coding strand of *hisA;* PAM: 5′-CGG-3′. spacer 1	TTGCACCTGGTGGATCTGAC	NA
pMSL45	Type IV-A1 crRNP; Protospacer is located on the coding strand of *hisA;* PAM: 5′-AAG-3′	CGTGGCAGCGGGTCGTTACCGTAATCGCGTTG	NA
pMSL46	Type IV-A1 crRNP; Protospacer is located in the histidine operon promoter*;* PAM: 5′-AAC-3′	GGTTCAGACAGGTTTAAAGAGGAATAAGAAAA	NA
pMSL47	Type IV-A1 crRNP; Protospacer is located within an internal promoter of the histidine operon on *hisC;* PAM: 5′-AAG-3′	CCTCCAGCGCAGTGTTTAAATCTTTGTGGGAT	NA
pMSL55	dCas9; Protospacer is located on the non-coding strand of *hisA;* PAM: 5′-CGG-3′. spacer 2	CCGGCATTAGATTTAATCGA	NA
pMSL56	dCas9; Protospacer is located on the non-coding strand of *hisA;* PAM: 5′-CGG-3′. spacer 3	TACGGCAAACAACGCGATTA	NA
pMSL57	dCas9; Protospacer is located on the non-coding strand of *hisA;* PAM: 5′-CGG-3′. spacer 4	GTTCCAGTGCAGGTTGGTGG	NA
pMSL70	dCas9; Protospacer is located on the coding strand of *hisA;* PAM: 5′-CGG-3′	TAATCCTGTAAGCGTGGCAG	NA
pSR106	Negative control in the genomic CRISPRi assays in *P. oleovorans* strains	ATGTGACTCTCCTCCGAAGAGGAGGTAAGTAC	BseRI

Plasmid	Description	Spacer sequence (5′-3′)	Restriction recognition sites for insert
pSR112	Type IV-A1 crRNA; Protospacer is located on the coding strand of *sfgfp* inserted in *P. oleovorans;* PAM: 5′-AAG-3′	GTGATGCGACCAACGGTAAACTGACCCTGAAA	NA
pSR121	Type IV-A1 crRNA; Protospacer is located on non-template strand in pBAD promoter of *sfgfp* inserted in *P. oleovorans;* PAM: 5′-AAG-3′	ATTAGCGGATCCTACCTGACGCTTTTTATCGC	NA
pSR122	Type IV-A1 crRNA; Protospacer is located on the coding strand of *sfgfp* inserted in *P. oleovorans;* PAM: 5′-AAG-3′	GCAGCCACCATCATCATCACCATTAAGCTGAA	NA
pSR124	Type IV-A1 crRNA; Protospacer is located on non-template strand 115 bp upstream of beginning of *sfgfp* inserted in *P. oleovorans;* PAM: 5′-AAG-3′	TCCACATTGATTATTTGCACGGCGTCACACTT	NA
pSR125	Type IV-A1 crRNA; Protospacer is located on non-template strand 58 bp downstream of end of *sfgfp* inserted in *P. oleovorans;* PAM: 5′-AAG-3′	GGCGGGGTTTGTTCTTCTTCGGGTTTACGCTT	NA
pSR129	Type IV-A1 crRNA; Protospacer is located on non-template strand 2421 bp upstream of beginning of *sfgfp* inserted in *P. oleovorans;* PAM: 5′-AAG-3′	CCAAGCGGGCTCGGGCGGCTTGTTGATGATCG	NA
pSR150	Type IV-A1 crRNA; Protospacer is located on coding strand 541 bp upstream of beginning of *sfgfp* inserted in *P. oleovorans;* PAM: 5′-AAC-3′	CCTGCAGCATGTGTGCAGGTTTGATCGTGCAT	NA
pSR151	Type IV-A1 crRNA; Protospacer is located on non-coding strand 555 bp upstream of beginning of *sfgfp* inserted in *P. oleovorans;* PAM: 5′-AAC-3′	CTGCACACATGCTGCAGGGTTCCAGGGTGACG	NA
pSR152	Type IV-A1 crRNA; Protospacer is located on coding strand 405 bp downstream of end of *sfgfp* inserted in *P. oleovorans;* PAM: 5′-AAG-3′	GCTATGTCTGAGTTAACTGTTGCATATCCCTA	NA
pSR153	Type IV-A1 crRNA; Protospacer is located on non-coding strand 418 bp downstream of end of *sfgfp* inserted in *P. oleovorans;* PAM: 5′-AAG-3′	AGGCGTTGGGGTATAGGATATGCAACAGTTA	NA

Plasmid	Description	Spacer sequence (5′-3′)	Restriction recognition sites for insert
pSR154	Type IV-A1 crRNA; Protospacer is located on coding strand 1183 bp upstream of beginning of *sfgfp* inserted in *P. oleovorans;* PAM: 5′-AAG-3′	TATTGGCAGATCAATGGCCAGGCCTGGGATAT	NA
pSR155	Type IV-A1 crRNA; Protospacer is located on template strand 88.828 bp upstream of beginning of *sfgfp* inserted in *P. oleovorans;* PAM: 5′-AAG-3′	TAAAATCAAAGCAAAAGTTATTGCAAGATCAC	NA
pSR160	Type IV-A1 crRNA; Protospacer is located on template strand 2435 bp upstream of beginning of *sfgfp* inserted in *P. oleovorans;* PAM: 5′-AAG-3′	CCGCCCGAGCCCGCTTGGCTTAGGGGATTCGT	NA
pSR162	Type IV-A1 crRNA; Protospacer is located on coding strand 3460 bp upstream of beginning of *sfgfp* inserted in *P. oleovorans;* PAM: 5′-AAG-3′	TCGCGCTCGGCGATCATGACCGCCCAGAGCCA	NA
pSR24	Type IV-A1 crRNA; Protospacer is located on pSR14; 5′-AAG-3′ PAM	CATCCAAGTTACGCATCAGATTCGAGACGCGA	NA
pSR54	Type IV-A1 crRNA; Protospacer is located on the coding strand of *lacZ* in BL21-AI*;* PAM: 5′-AAG-3′	ATCGCACTCCAGCCAGCTTTCCGGCACCGCTT	NA
pSR56	Negative control Type IV-A1 in the genomic *lacZ* CRISPRi assays	ATGTGACTCTCCTCCGAAGAGGAGGTAAGTAC	BseRI
pSR77	Negative control used in the histidine auxotrophic Type IV-A1 CRISPRi assays	ATGTGACTCTCCTCCGAAGAGGAGGTAAGTAC	BseRI

Supplementary Table S8. Primers used for RT-qPCR.

**Oligo name**	**Sequence (5'-3')**	**Primer Efficiency Test**
qPCR *hisA* fw	CGTGGTACGTCTCCATCAGG	Efficiency: 93%<br>R^2: 098
qPCR *hisA* rv	GCGGGATTTGACGTTTAGCC	
qPCR *hisH* fw	CTGTTTTTACCCGGCGTTGG	Efficiency: 98%<br>R^2: 0.99
qPCR *hisH* rv	CCCAGCAGTTGCATCCCTAA	
qPCR *hisF* fw	AGAAGGTGCAGACGAACTGG	Efficiency: 91.6%<br>R^2: 0.95
qPCR *hisF* rv	GAGACTTAATCCCACCCGCC	
qPCR *recA* fw	GTTCCATGGATGTGGAAACC	Efficiency: 92%<br>R^2: 0.99
qPCR *recA* rv	ATATCGACGCCCAGTTTACG	

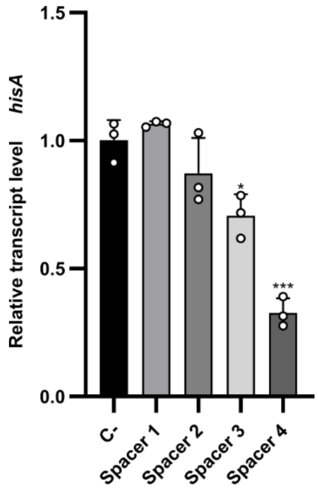

Supplementary Figure S1. RT-qPCR of different spacers tested for dCas9-mediated CRISPRi on *hisA* gene. Different spacers for the dCas9-mediated CRISPRi on target (3) were tested. RT-qPCR experiments were performed with n=3 independent colonies. A non-targeting crRNA was used as a negative control (C-). Statistical analysis was performed in comparison to C- using an unpaired two-tailed t-test. Data represent the mean (± SD) with * p = 0.0114 and *** p = 0.0003.

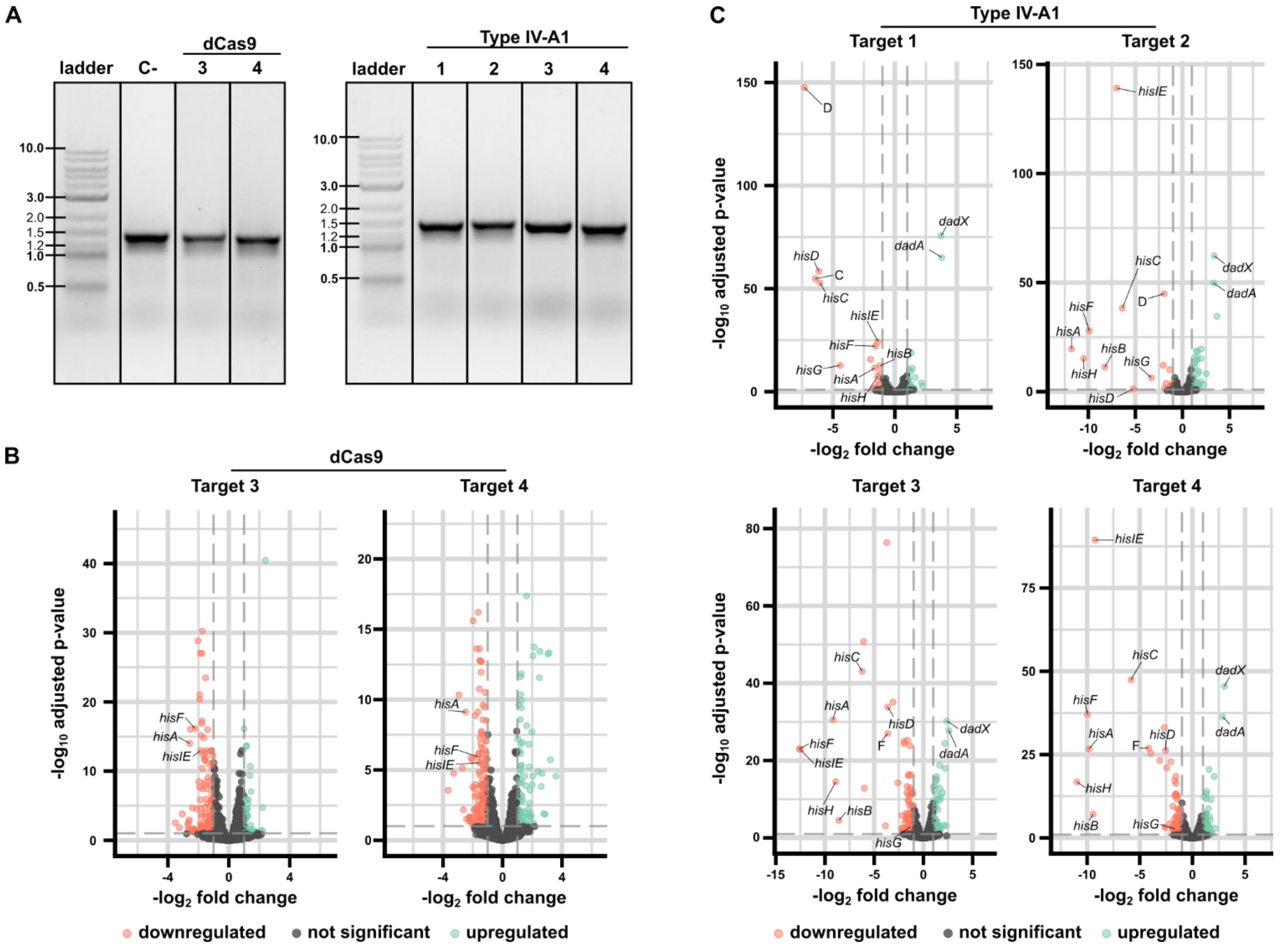

Supplementary Figure S2. Analysis of CRISPRi transcriptome effects. A. Agarose gel showing the result of a PCR analysis testing DNA integrity after CRISPRi assays using dCas9 or Type IV-A1. C-: Non-targeting control. Ladder: 1 kb Plus (NEB) **B.** Volcano plots showing the differential expression of genes after dCas9 treatment for the two indicated target sites**.** Significantly regulated genes are highlighted in salmon (downregulated) and green (upregulated). **C.** Volcano plots showing the differential expression of genes after Type IV-A1 treatment for the indicated target sites**.** Significantly regulated genes are highlighted in salmon (downregulated) and green (upregulated).

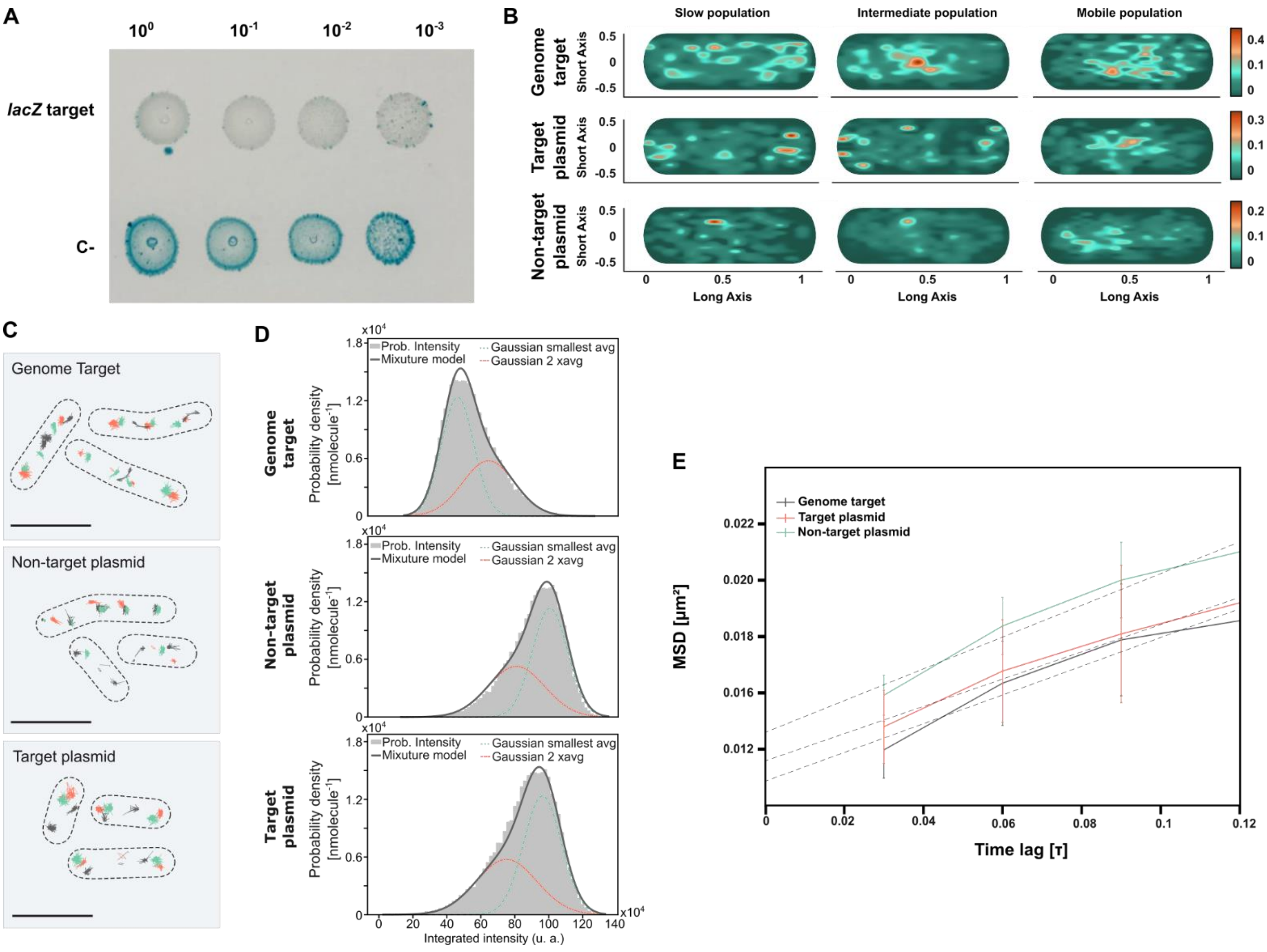

Supplementary Figure S3. Spatiotemporal dynamics of the mNeonGreen-tagged crRNPs with different targets. A. Blue-white screening after CRISPRi with mNeonGreen-tagged Type IV-A1 crRNPs targeting *lacZ*. **B.** Heat maps of the three different molecule populations for each condition. Tracks are projected onto a representative cell from each condition. Heat maps indicate the spatial distribution of mNeonGreen-tagged crRNPs heterologously expressed in *E. coli* BL21-AI. The yellow-reddish areas indicate the distribution of most of the tracks with longer scanning times. **C.** Projections of all tracks observed in three representative cells from the indicated conditions, assigned according to the diffusion coefficient to the slow (salmon), intermediate (gray), or mobile (green) population. **D.** Distribution density function of integrated spot intensities for each condition. Number of particles detected for: Genome target (58322), non-target plasmid (52987), and target plasmid (57329). In the best estimation, there are two populations of average integrated intensity for all conditions and are represented in arbitrary units (u.a). The two populations are shown as Gaussian distributions (salmon and green) and the mean number of particles is determined in the intersection point of both curves. **E.** Comparison of the Mean Square Displacement (MSD) of mNeonGreen-tagged crRNPs after different time intervals in the three conditions. Data points represent the mean MSD, with error bars indicating the standard error of the mean (SEM) (Genome target: 0.11 ± 0.018 μm^2; non-target plasmid: 0.14 ± 0.015 μm^2; target plasmid: 0.13 ± 0.017 μm^2).

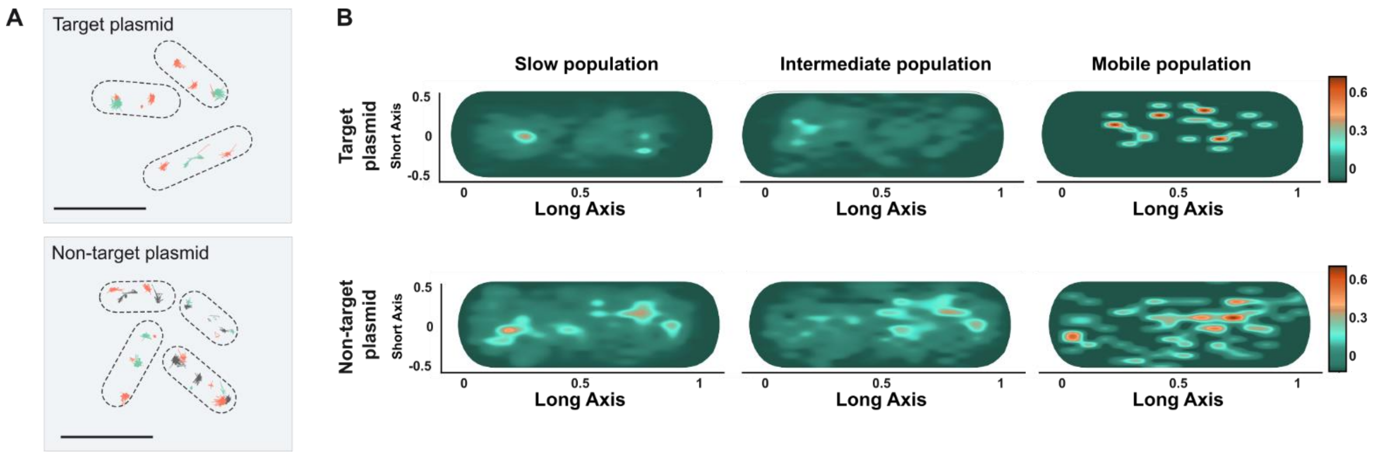

Supplementary Figure S4. Spatiotemporal dynamics of the DnaX-mScarlet protein in cells expressing mNeonGreen-tagged crRNPs targeting or not targeting a plasmid. A. Three representative cells from each condition that contain projections of all tracks, assigned according to the diffusion coefficient to the slow (salmon), intermediate (gray), or mobile (green) population. **B.** Heat maps of the three different molecule populations for each condition. Tracks are projected onto a representative cell from each condition. Heat maps indicate the spatial distribution of DnaX-mScarlet in *E. coli* BL21-AI. The yellow-reddish areas indicate the distribution of most of the tracks with longer scanning times.

SUPPLEMENTARY REFERENCES

Guo, X., Sanchez-Londono, M., Gomes-Filho, J.V., Hernandez-Tamayo, R., Rust, S., Immelmann, L.M., Schäfer, P., Wiegel, J., Graumann, P.L. and Randau, L. (2022) Characterization of the self-targeting Type IV CRISPR interference system in *Pseudomonas oleovorans*, *Nature Microbiology,* **7,** 1870–1878.

Kovach, M.E., Elzer, P.H., Steven Hill, D., Robertson, G.T., Farris, M.A., Roop, R. and Peterson, K.M. (1995) Four new derivatives of the broad-host-range cloning vector pBBR1MCS, carrying different antibiotic-resistance cassettes, *Gene,* **166,** 175–176.

Martínez-García, E. and Lorenzo, V. de (2011) Engineering multiple genomic deletions in Gram-negative bacteria: analysis of the multi-resistant antibiotic profile of Pseudomonas putida KT2440, *Environmental microbiology,* **13,** 2702–2716.

Silva-Rocha, R., Martínez-García, E., Calles, B., Chavarría, M., Arce-Rodríguez, A., Las Heras, A. de, Páez-Espino, A.D., Durante-Rodríguez, G., Kim, J. and Nikel, P.I. *et al.* (2013) The Standard European Vector Architecture (SEVA): a coherent platform for the analysis and deployment of complex prokaryotic phenotypes, *Nucleic Acids Research,* **41,** D666-75.
